# MyESL: A Software for Evolutionary Sparse Learning in Molecular Phylogenetics and Genomics

**DOI:** 10.1093/molbev/msaf224

**Published:** 2025-09-15

**Authors:** Maxwell Sanderford, Sudip Sharma, Glen Stecher, Michael Suleski, Jun Liu, Jieping Ye, Sudhir Kumar

**Affiliations:** Institute for Genomics and Evolutionary Medicine, Temple University, 1925 N. 12th Street, Philadelphia, PA 19122, USA; Institute for Genomics and Evolutionary Medicine, Temple University, 1925 N. 12th Street, Philadelphia, PA 19122, USA; Department of Biology, Temple University, 1900 N. 12th Street, Philadelphia, PA 19122, USA; Institute for Genomics and Evolutionary Medicine, Temple University, 1925 N. 12th Street, Philadelphia, PA 19122, USA; Institute for Genomics and Evolutionary Medicine, Temple University, 1925 N. 12th Street, Philadelphia, PA 19122, USA; Infinia ML Inc., 4309 Emperor Blvd, Durham, NC 27703, USA; Zhejiang Lab, 2880 Wenyi West Road, Hangzhou, Zhejlang 311100, P.R. China; Institute for Genomics and Evolutionary Medicine, Temple University, 1925 N. 12th Street, Philadelphia, PA 19122, USA; Department of Biology, Temple University, 1900 N. 12th Street, Philadelphia, PA 19122, USA

**Keywords:** phylogenomics, sparse learning, machine learning, molecular evolution

## Abstract

Evolutionary sparse learning uses supervised machine learning to build evolutionary models where genomic sites loci are parameters. It uses the Least Absolute Shrinkage and Selection Operator with bi-level sparsity to connect a specific phylogenetic hypothesis with sequence variation across genomic loci. The MyESL software addresses the need for open-source tools to perform evolutionary sparse learning analyses, offering features to preprocess input phylogenomic alignments, post-process output models to generate molecular evolutionary metrics, and make Least Absolute Shrinkage and Selection Operator regression adaptable and efficient for phylogenetic trees and alignments. The core of MyESL, which constructs models with logistic regressions using bi-level sparsity, is written in C++. Its input data preprocessing and result post-processing tools are developed in Python. Compared to other tools, MyESL is more computationally efficient and provides evolution-friendly inputs and outputs. These features have already enabled the use of MyESL in two phylogenomic applications, one to identify outlier sequences and fragile clades in inferred phylogenies and another to build genetic models of convergent traits. In addition to the use in a Python environment, MyESL is available as a standalone executable compatible across multiple platforms, which can be directly integrated into scripts and third-party software. The source code, executable, and documentation for MyESL are openly accessible at https://github.com/kumarlabgit/MyESL.

## Introduction

Understanding the genetic basis of organismal relationships and biological traits is a key goal in evolutionary biology. Evolutionary sparse learning (ESL) is a comparative sequence analysis framework that uses supervised machine learning with a sparsity constraints to develop genetic models of evolutionary hypotheses (Kumar and Sharma 2021; Kumar et al. 2024; Sharma and Kumar 2024; Allard et al. 2025). Evolutionary hypotheses can be created based on whether sequences belong to a specific phylogenetic clade or if a trait is present or absent in organisms. In ESL models, parameters are sites and genomic loci, such as genes, proteins, exons, introns, and intergenic regions. ESL applies the Least Absolute Shrinkage and Selection Operator (LASSO) (Tibshirani 1996), which automatically compares alternative models that involve different combinations of genomic loci and positions using sparse group LASSO with logistic loss (Simon et al. 2013; Qiao et al. 2017; Kumar and Sharma 2021). The resulting ESL model includes positions and loci harboring variation concordant with the evolutionary hypothesis, along with measures of the importance of positions and loci (Kumar and Sharma 2021). The ESL model is a statistical equation that predicts whether a new sequence belongs to the specific clade or if a trait is present or absent in an organism based on its genotype, a feature missing from conventional methods of molecular phylogenetics and evolution.

Using ESL, novel approaches and metrics have been developed to detect outlier sequences and fragile clades in organismal phylogenies inferred from phylogenomic alignments (Sharma and Kumar 2024). Unlike conventional methods, ESL can identify gene-species combinations that significantly influence the fragility of an inferred clade, without requiring prior knowledge of alternative hypotheses or using base substitution models (Sharma and Kumar 2024). ESL-based metrics complement classical approaches, such as maximum likelihood methods, which may require computationally intensive comparisons and alternative phylogenetic hypotheses, e.g. (Brown and Thomson 2016; Shen et al. 2017). In another application, ESL was used to develop genetic models of convergent traits where species with contrasting traits were chosen from each clade (Allard et al. 2025). The ESL with Paired Species Contrast (ESL-PSC) approach revealed the excess of convergent amino acid substitutions in hearing-related proteins of mammals with echolocation (Allard et al. 2025). Analyses of computer-simulated datasets established that ESL-PSC can be more effective than traditional methods at identifying proteins harboring convergent substitutions in species that have acquired the convergent trait (Allard et al. 2025).

For both of these ESL applications, the MyESL software suite was used to perform sparse group LASSO regression with logistic loss. Sparse group LASSO introduces bi-level sparsity by organizing features (sites) into user-defined mutually exclusive groups (genes), with sparsity enforced both within and among groups (Tibshirani 1996; Simon et al. 2013; Kumar and Sharma 2021). ESL was initially implemented using the Sparse Learning with Efficient Projections (SLEP) package in MATLAB (Liu et al. 2011; Kumar and Sharma 2021). However, MATLAB is neither universally accessible nor free, which limited ESL's usability. For proprietary reasons, the MATLAB version of ESL could not be bundled with third-party tools like MEGA (Kumar et al. 2024). Packaging modified MATLAB code into standalone software requires a MATLAB compiler, which is also not freely available.

Additionally, available open-source R and Python packages either lack a sparse group lasso implementation or are not computationally efficient for genome-scale sequence data with millions of features (Yang and Zou 2015; Zeng and Breheny 2017; Klosa et al. 2020; Civieta et al. 2021; Liang et al. 2024). For example, *sparsegl* could not complete calculations for a phylogenomic dataset containing DNA sequences of 63,430 (63K) loci from 363 bird species (Stiller et al. 2024) on a workstation with 64 GB of RAM (54 GB available) and eight physical cores. At most, a subset of data with 10,000 loci could be analyzed within a reasonable time, which used ∼48 GB of RAM. We estimated that *sparsegl* would need 302 GB of RAM to analyze the full dataset (see *Results*). Besides the high computational demand, this and all other existing packages lack built-in support for input data preprocessing and model post-processing specific to phylogenomics and functional genomics.

Below, we introduce MyESL, an open-source software that implements bi-level sparse group LASSO and related features in C++ for efficiency. It also includes a new library of key input/output functions to process phylogenetic trees and sequence alignments. This library is written in Python, a language commonly used in genomics research. These programming language choices for our MyESL package enable compilation into platform-specific executables (e.g. Microsoft Windows and macOS), which can be distributed and utilized without setting up a Python environment. These programs enabled ESL analyses to build genetic models of evolutionary hypotheses, providing genes and sites along with their importance in the genetic model, and predicting evolutionary characteristics of any species using the genetic model. The application of genetic models and other metrics can be useful in various contexts, including two recent applications mentioned above. MEGA software version 12 (Kumar et al. 2024) already makes the MyESL application with *DrPhylo* accessible through MEGA's graphical user interface for identifying highly influential genes and sequences within species clades (Sharma and Kumar 2024).

## Results

MyESL begins ESL analysis by preprocessing input data, including sequence alignments and hypotheses of sequence classes or a phylogenetic tree. These processes are controlled using different options through the command line.

### One-Hot Encoding of Sequence Alignment

**Fig. 1.**
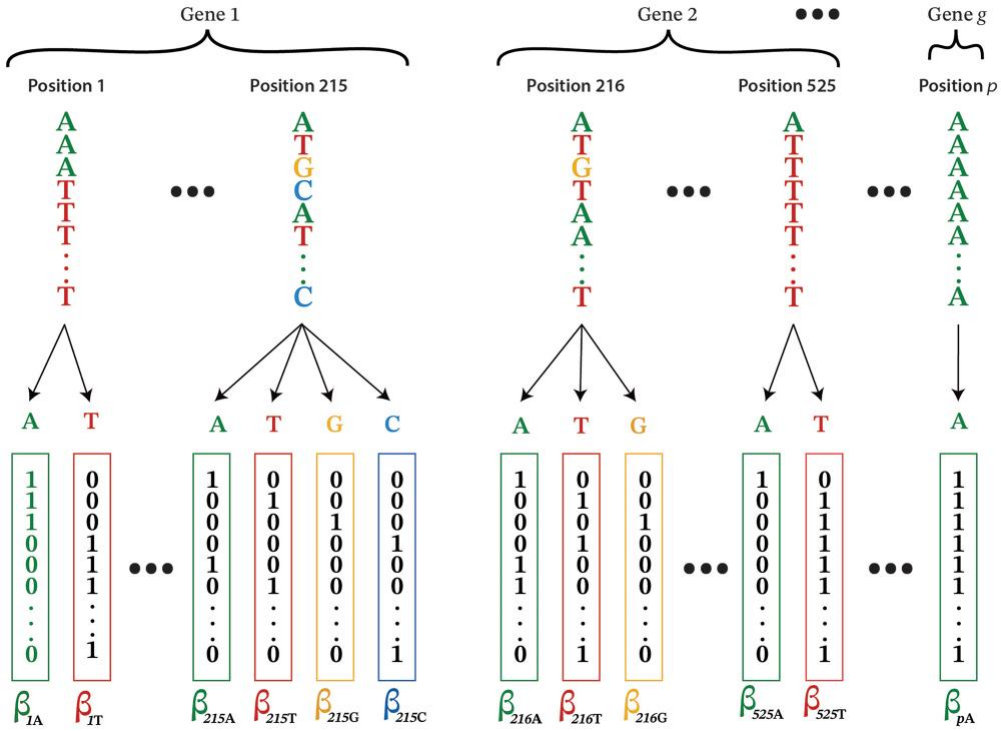
**One-hot encoding and evolutionary sparse learning.** The sequence alignment input to ESL consists of *p* positions (columns) belonging to *g* groups (e.g. genes). The one-hot representation of the alignment is shown below the sequence, where each allele present at a position gets a bit column containing a 1 when the given allele is present in the position and a zero otherwise. Each bit column serves as a feature in the ESL modeling, which assigns weights (β) to each one. β captures the correlation between the binary pattern in the bit column and the evolutionary hypothesis in which taxa (rows in the alignment) are labeled as +1 or −1.

MyESL starts by reading sequence alignments in FASTA format. A list of FASTA files (one per line) is provided through a text file as the initial input to MyESL. Sequence alignments are loaded from these FASTA files. MyESL assumes that each FASTA file represents a separate group of alignment positions, such as a gene or a set of sites meant to be treated as a group (Fig. 1). All sequences are converted numerically using binary one-hot encoding (Fig. 1), a feature implemented in C++ to maximize input data processing speed. In one-hot encoding, each position in the sequence alignment is represented by as many bit columns as there are unique characters at that position (Fig. 1). No bit columns are created for the alignment gap character (e.g. –) or the missing data character (e.g. ?).

A ***--data_type***  *<nucleotide>* option directs MyESL to recognize A, T, C, G, and U as valid bases regardless of case. All other characters will be treated as missing data (similar to “?”). Likewise, the *<protein>* option considers all unambiguous IUPAC amino acid letters (case-insensitive) as valid residues. The *<molecular>* option allows the use of both nucleotide and amino acid letters as valid, enabling a mixture of the two data types. If none of these options are specified, MyESL will treat all letters (case-sensitive) and digits as distinct characters, which is useful for analyzing different types of data. For example, users might include information about methylation status, presence or absence of genes or genomic segments, and other features in their input data files. Non-molecular characteristics and features could be specified using letters and digits, allowing ESL analyses. When using this flexibility, users should prepare their inputs cautiously and interpret the results carefully.

MyESL compresses the resulting data matrix by removing all monomorphic sites in organisms selected for ESL analysis. All sequences at that position share the same character across taxa, except for missing data and alignment gaps. Singleton bit columns are also eliminated because only one alignment row has an unambiguous character state different from the others. Such features are generally not informative in the LASSO regression analysis. However, users can choose to keep singleton sites using the ***--include_singletons*** option. To further reduce the number of features for LASSO, one can choose to remove all bit columns where bit 1 appears fewer than a specified number of times (***--bit_ct***  *<count>*), which can help decrease memory requirements for ESL analysis of extremely long sequences.

### Reading and Processing the Evolutionary Hypothesis

The evolutionary hypothesis in MyESL can be specified in two ways: ***--classes***  *<classfile.txt>* or ***–tree***  *<file_name.nwk>*. The *classfile.txt* is a tab-separated text file with two columns: the organism name in the first column and the class designation (+1 or −1) in the second, which indicates responses (outcomes) for LASSO regression. The +1 class is the focal class in MyESL, which can be a clade in a phylogeny or a trait of interest. Alternatively, MyESL can automatically generate class designations by analyzing an input phylogeny, which should be a rooted tree in the Newick format. In this tree, the internal node (clade) with a label will serve as the focal clade. All taxa within the subtree defined by that internal node are assigned to the +1 class. The remaining taxa in the tree are assigned to the −1 class. If multiple nodes in the input phylogeny have labels, the ***--clade_list***  *<filename.txt>* option specifies the focal clades for analysis. Otherwise, MyESL will build an ESL model for each labeled clade iteratively. MyESL can also perform multiple ESL analyses by automatically generating labels for all internal nodes in the phylogeny using the ***--gen_clade_list*** option, where users can set the minimum and maximum number of clade members with ***--cladesize_cutoff_lower***  *<int>* and ***--cladesize_cutoff_upper***  *<int>*, respectively. Clades with fewer or more members than the specified range are excluded, and MyESL will not build clade models for them.

### Class Balancing

The number of organisms assigned a +1 and −1 may be unequal, leading to a class-imbalanced dataset. Class imbalance can significantly impact the performance of supervised machine learning models. Specifically, when one class (e.g. taxa within the focal clade) is underrepresented, the model may become biased toward predicting the majority class, resulting in low classification probabilities for species in the minority class. This is discussed in the section below, which demonstrates the use of MyESL. Therefore, MyESL provides several methods to achieve class balancing, which involves selecting an equal number of taxa in both classes (+1 and −1) using the ***--class_bal*** option. One can either upsample the minority class (***--class_bal***  *<up>*) or downsample the majority class (***--class_bal***  *<down>*) to attain balance. Upsampling is done by randomly sampling species *with replacement* until the minority class reaches the same number of species as the majority class, meaning the same species may be included multiple times. Conversely, downsampling involves sampling from the majority class without replacement. In these cases, the taxa included are chosen randomly. The ***--class_bal***  *<weighted>* option assigns weights inversely proportional to class size to balance the contributions of both classes during model building, so it does not require random subsampling of the majority class.

MyESL also offers a new phylogeny-aware class balancing option (***--class_bal***  *<phylo>*) when the evolutionary hypothesis is provided through a Newick tree. In this method, MyESL first assigns a +1 to all taxa in the focal clade. It then examines the sister group of the focal clade and assigns −1 to each taxon there (i.e. first cousins). If the number of taxa in the −1 class is smaller than in the focal clade, the search continues upward by moving to the ancestor of the focal clade and assigning taxa in the sister group at the next level to be −1 (i.e. second cousins). This process repeats until no more taxa are available or the number of taxa labeled −1 exceeds those in the focal clade. At that point, if the two classes have an unequal number of taxa, MyESL iteratively removes taxa from the larger class, starting with the most distant taxa from the focal clade, until both classes contain the same number of taxa. Phylogenetically aware class balancing excludes taxa that are evolutionarily distant from the focal clade. Therefore, when the number of taxa in class +1 exceeds that in class −1, this approach should be used carefully, as removing taxa from the focal clade could distort the hypothesis being tested. In such cases, weighted class balancing is a better option.

### Model Building Using LASSO Regression

MyESL estimates the coefficients of the sparse group LASSO regression model by minimizing the logistic loss (Liu et al. 2011), which is defined as:


(1) $$L^{\prime }(\beta )=l(\beta )+\lambda_{1}|\beta {|}_{1}+\lambda_{2}\Sigma_{g}w_{g}|\left|\beta_{g}\right|{|}_{2}$$

Here, the first term is the logistic loss function, and the second is the penalty for including individual bit columns in the regression model. *λ_1_*, the regularization parameter, penalizes the inclusion of bit columns in the ESL model. *β* is the column vector of regression coefficients (see Fig. 1), with its norm $\left|\beta |=\Sigma_{i=1}|\beta_{i}\right|$ where *i* ranges from 1 to the number of bit columns in the entire dataset. The third term penalizes the inclusion of groups into the regression model. Here, *λ_2_* is the group regularization parameter, and $\beta_{g}=\Sigma_{i=1}|\beta_{gi}|$, where *i* ranges from 1 to the number of bit columns in group *g*, and *β_gi_* is the regression coefficient for the *i-th* feature in group *g*. The product of *β_g_* and the group weight (*w*_g_) is summed over all *G* groups.

The group weight is usually the square root of the number of bit columns in group *g*, which MyESL assumes. Alternatively, users can provide group weights through a text file (***--group_wt***  *<filename.txt>*) that contains two tab-separated columns: the first with group names and the second with the corresponding group weights. Both bit columns and group penalty parameters, *λ*1 and *λ*2, are specified by users using the arguments ***--lambda1***  *<float>* and ***--lambda2***  *<float>*. The values of these parameters range from 0 to 1, with a default of 0.1 for both. MyESL also supports performing LASSO regression without group sparsity by using the ***--no_group_penalty*** option or by providing only a single FASTA file as data input.

### Sparse Group LASSO Implementation

The LASSO regression analysis in MyESL is implemented in C++ for efficiency and portability across various platforms. The C++ source code directly translates the MATLAB code, *sgLogisticR*, used for logistic regression, along with other SLEP functions from Kumar and Sharma (2021). Regression models optimize the regression weights using Moreau-Yosida regularization (Liu and Ye 2010; Liu et al. 2011) and minimize the logistic loss for sparse group LASSO regression. MyESL employs the Armadillo library in C++ for linear algebra and scientific computing (Sanderson and Curtin 2016), which can utilize multiple cores for matrix operations.

### ESL Models, Cross-Validation, and Classification

MyESL uses supervised machine learning to develop an ESL model based on a given hypothesis. In this process, the ESL estimates regression coefficients *β* for each bit position in the one-hot encoded matrix, where most of these coefficients will be zero (*β* = 0) due to the sparsity constraint. These *β* coefficients can be further optimized through cross-validation when using the ESL model for classification purposes. Cross-validation in MyESL is performed on the training dataset with the option ***–kfold***  *<int>*. For example, setting k-fold = 5 means dividing all taxa in the dataset into five subsets, each with balanced representation. *k*–1 subsets (80% of the data) are used for training, while the remaining subset (20%) is held out for testing. MyESL generates feature weights from *k*–1 subsets and calculates classification accuracy on the holdout sample.

MyESL offers a separate pipeline (*MyESL_model_apply.exe*) for using the pretrained ESL model, specifically the *β* coefficients, to classify a new set of taxa (see the section: *An Example demonstrating the use of MyESL*). This function can classify new organisms whose sequences are aligned with the data used to build the model. Each organism in the test set receives a prediction score (*SPS*) and a probability (*SPP*; ranging from 0 to 1), both output in a tab-separated text file. A prediction score ≥ 0 and a predicted probability ≥ 0.5 indicate classification into the class labeled +1, representing a species group or taxon with the trait.

### Building Multiple ESL Models

Building multiple models with the same feature and response data is common in machine learning to find the best pair of sparsity parameters or to enable model averaging. MyESL allows users to generate multiple ESL models by performing a grid search over the regularization parameter space. The grid search option lets users set the *λ* parameters for bit (***--lamba1_grid***  *<float, float, float>*) and group sparsity (***--lamba1_grid***  *<float, float, float>*) by defining the minimum [0–1], maximum [0–1], and step size [0–1] of the parameter space. Users can choose optimal lambda parameter pairs by setting thresholds for root mean square error (RMSE) with ***--grid_rmse_cutoff***  *<float>*, which helps filter out overfitted or underfitted models by keeping those with low RMSE values (|Allard et al. 2025). The default RMSE cutoff is set to 100, resulting in the retention of all models from the applied *λ* combinations. Similarly, ***--grid_acc_cutoff***  *<float>* assists in selecting models that meet a minimum training classification accuracy, with a default of 0.95. The ***--min_groups*** <*int*> option sets a lower limit on the number of groups included in the model, which helps control sparsity (Sharma and Kumar 2024). These criteria can produce multiple equally valid models, all of which can be used for prediction and further analysis.

MyESL processes the regression coefficient and generates a series of result files that contain different sparsity scores. These are tab-separated text files created using the following option: **--*stats*_*out***  *<PGHS>*. Other letters in the input string for the option will yield the corresponding results as follows: P: Position Sparsity Scores; G: Group Sparsity Scores (*GSS*); H: Hypothesis Sparsity Scores (*HSS*); S: A file containing both Sequence Prediction Score (*SPS*) and Sequence Prediction Probability (*SPP*) (see details in Kumar and Sharma (2021)).

### An Example Demonstrating the Use of MyESL

**Fig. 2.**
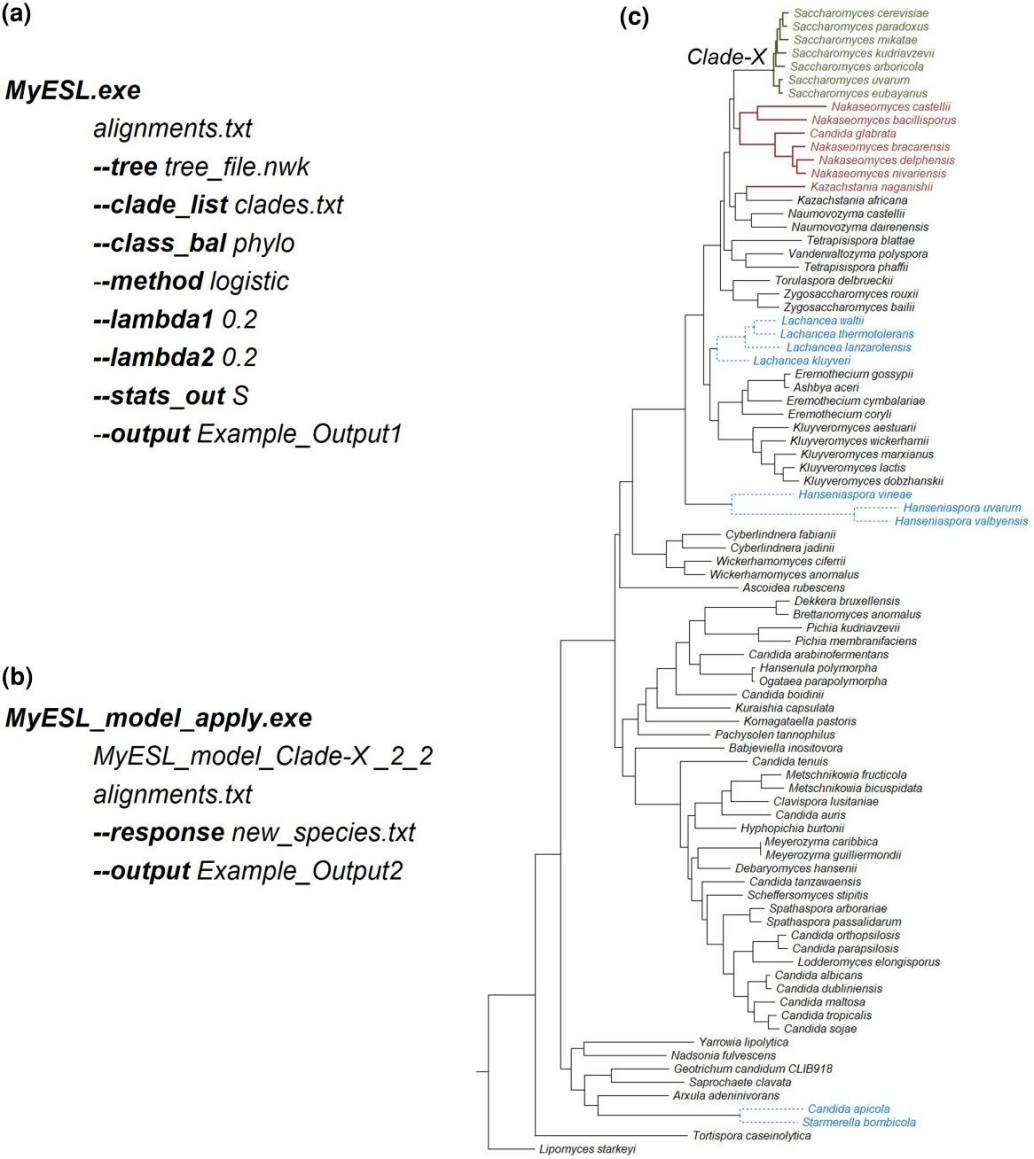
**An example demonstrating the use of MyESL for building a genetic model for a clade.** Command lines to (*a*) construct the genetic model using MyESL and (*b*) apply it to test if sequences not used for training belong to the clade. (c) The maximum likelihood phylogeny of 86 fungal species inferred from concatenated alignments of 1,233 nuclear proteins (see text). The focal clade includes species with names in green (numerically encoded as +1). Species with names in red are negative controls (numerically coded as −1), which were selected automatically by the phylogeny-aware class balancing in MyESL. Species with names in blue were used for making predictions about membership of Clade-X using the command in panel *b*.

We provide an example of using MyESL to build a clade model from a phylogenomic dataset consisting of 1,233 proteins from 86 fungal species previously analyzed in Sharma and Kumar (2024). A clade model was built for a group of seven species from the *Saccharomycetaceae* family (green; Fig. 2c). The Clade-X received 100% standard bootstrap support in a maximum likelihood analysis of the concatenated sequence alignment (Shen et al. 2016, 2017). Because the focal clade contains only seven species, which is significantly fewer than the 79 species outside it, classes are imbalanced. To address this, we applied phylogenetically aware class balancing, selecting seven of the closest sister species to the focal clade (red; Fig. 2c).

The MyESL command line used for this analysis is shown in Fig. 2a (see Supplementary material, Table S1 for the current list of options). All the files containing sequence alignments are listed in *alignments.txt* (one per line), and the species tree is provided in the Newick format (*tree_file.nwk*). Clades for which ESL analysis is desired are specified in *clades.txt*; it only contains “Clade-X” for the current example. The command line directs MyESL to build a model using sparse group Lasso with logistic loss, with site and group penalties of 0.2 each. Option ***--stats_out***  *S* generates a tab-separated text file with *SPS* and *SPP* scores for all species in the clade model. *SPS* and *SPP* are calculated using formulas explained in Kumar and Sharma (2021).

MyESL outputs include *SPS*, *SPP*, and a grid image of the model, stored in the *Example_Output1* directory. For the seven species within the focal clade, *SPS* values ranged from 1.3 to 1.4, and *SPP* values from 0.78 to 0.80, indicating higher confidence in classification. For comparison, we also built a clade model without class balancing (removing the option ***--clade_bal***  *phylo*). In this case, *SPS* values for the focal clade species decreased to 0.11 to 0.14 (*SPP* values became 0.52 to 0.53), suggesting a lack of confidence in correct classification without class balancing.

MyESL also creates a model file named *MyESL_model_Clade-X_2_2.txt* for the ESL analysis with class balancing, which contains estimated beta coefficients for all binary one-hot encoded columns derived from individual sites and included in the final model. This file is saved in the *Example_Output1* directory. We selected nine fungal species (blue, Fig. 2c) whose sequence alignments were aligned with those used in model training. The commands and options for this analysis are shown in Fig. 2b. The file *new_species.txt* contains a list of species for which predictions are made, with one species name per line. The analysis generates a tab-separated text file and an image of the model grid. The text file reports the predicted *SPS* and *SPP* values for each new species. The *SPS* values for these species range from –0.19 to –0.73, with *SPP* values from 0.32 to 0.45. *SPS* < 0 and *SPP* < 0.5 indicate that these species are not members of Clade-X, consistent with their positions in the fungal phylogeny.

### Computational Efficiency of MyESL

**Fig. 3.**
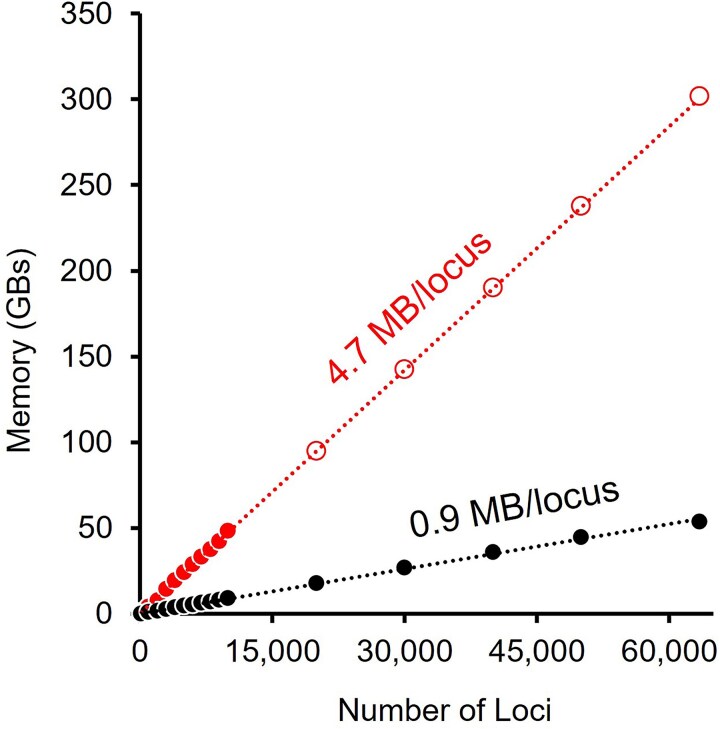
**Computational efficiency of MyESL.** Comparison of the RAM usage (in gigabytes) for conducting sparse learning with group LASSO for data subsets with varying numbers of loci using MyESL (black) and *sparsegl* in R (red). For *sparsegl*, filled circles represent RAM usage in actual data analysis, while open circles represent the projected RAM based on the linear regression through the origin. The RAM usage for MyESL analysis is based on data analysis performed on a computer with 54 GB of RAM available for use.

We compared the performance of MyESL with *sparsegl*, an R package for sparse group LASSO (Liang et al. 2024), using the Birds dataset described in the *Introduction*. Specifically, we evaluated the time and memory requirements for building an ESL model. All conserved sites and singletons were removed when FASTA files of sequence alignments were converted into binary one-hot encoded feature files using MyESL for all the following analyses. During model building, memory usage for *sparsegl* in R increased linearly at the rate of ∼4.7 GB per 1,000 loci (Fig. 3, filled red circles). In comparison, MyESL used only 0.9 GB per 1,000 loci, which is five times less than *sparsegl* (Fig. 3, filled black circles). Based on these trends, we estimated that analyzing all 63K loci would require 302 GB for *sparsegl*, which was not feasible on the computer system with 64 GB of RAM. In contrast, MyESL required 53.8 GB to analyze all 63,430 loci (73,425,979 bit columns). MyESL completed the analysis in 40 minutes (wall time).

### Distributions

The Python and C++ source codes for all custom functions used in MyESL are openly available on the GitHub repository: https://github.com/kumarlabgit/MyESL. The repository provides instructions for installing MyESL in a Python environment on Linux and for performing MyESL analysis on a clade within an example phylogeny using empirical sequence alignments. We have also bundled these MyESL utilities into a standalone Windows executable (.exe) file named *MyESL.exe*, which is distributed through the same GitHub repository. Using this executable, we connected MyESL with *DrPhylo* mode to MEGA 12 (Kumar et al. 2024) via its AppLinker interface, allowing MyESL's functions to be easily accessed by users with a single click when viewing the inferred phylogeny in MEGA's Tree Explorer.

## Supplementary Material

msaf224_Supplementary_Data

## Data Availability

The phylogenomic datasets used in this study are publicly accessible. The Fungi dataset was sourced from Shen et al. (2017), and the Birds dataset from Stiller et al. (2024) . All input and output files related to Figs. 2a and 2b are provided in the *GitHub* (https://github.com/kumarlabgit/MyESL).
