# Supplementary material for: MyESL: A Software for Evolutionary Sparse Learning in Molecular Phylogenetics and Genomics
Source: Mol Biol Evol. 2025 Sep 15;42(10):msaf224. doi: 10.1093/molbev/msaf224 (PMC12498521; doi:10.1093/molbev/msaf224)
Supplement: msaf224_Supplementary_Data [file msaf224_supplementary_data.pdf]

Supplementary Table

A list of MyESL options and associated descriptions. There are two commands: MyESL.exe (MyESL in Python) for building the ESL model and MyESL_model_apply.exe (MyESL_model_apply in Python) for applying the ESL model to prediction. All of these options are consistent in both the executable and Python environments.

Option	Description
**Model Options**	
<file name>	A text file containing full paths for all gene sequence alignments in FASTA format.
**--tree** <tree file name>	A phylogenetic tree in the Newick format with clade labels. If the response file is not provided using the text format, MyESL processes the text file to create the response for the ESL model.
**--clade_list**<br><text_file>	A text file containing the list of all clades for which clade models are built. Each line in the file contains clade labels.
**--gen_clade_list**<br><lower, upper>	For use when the input phylogeny does not contain any clade names. It automatically generates clade labels for all internal nodes whose descendant species count is within the specified **lower** and **upper** bounds. By default, both values are set to 0, which results in the assignment of clade labels to **all internal nodes** in the phylogeny.
**--auto_name_length**<br><int>	Specifies the number of characters to extract from species names when generating internal node labels for clades. The default is **3**, meaning the first three letters of species names will be used to construct clade labels.
**--clade_size_cutoff_lower**<br><int>	It enables users to automatically generate candidate clades for model building by specifying a lower and upper bound on clade size (both numeric). Clades containing species within the specified size range will be selected, and ESL models will be built for each of them. ESL models will not be built for other clades containing species more or fewer than the specified cut-off.
**--clade_size_cutoff_upper**<br><int>	
**--class_bal**<br><method>	For selecting the strategy for class balancing:<br>● **up**: Upsampling the smaller class to match the size of the larger class.<br>● **down**: Downsampling the larger class to match the smaller class<br>● **weighted**: Applying inverse class weights during model training<br>● **phylo**: Phylogeny-aware balancing that selects training samples based on evolutionary relationships
**--method**<br><method>	Specifies the type of loss function or regression to be used in model training. The default option is logistic.<br>● logistic: which performs sparse group lasso or lasso regression with logistic loss and is suitable for binary response variables (e.g., +1/–1).

Option	Description
	• leastr: applies sparse group lasso or lasso regression using least squares loss, and should be used when the response variable is continuous, ranging from $-\infty$ to $+\infty$.
**--lambda1**<br><float [0-1]>	Specifies the site sparsity penalty parameter that controls the inclusion of features (e.g., sites) in the clade model. The value must be a float between 0 and 1, where higher values apply stronger penalization, resulting in a sparser model that selects fewer sites.
**--lambda2**<br><float [0-1]>	Specifies the group sparsity penalty parameter that controls the inclusion of feature groups (e.g., genes) in the clade model. The value must be a float between 0 and 1, where higher values apply stronger penalization, resulting in a sparser model that selects fewer groups.
**--no_group_penalty**	Enables monolevel Lasso analysis by disabling the group-level sparsity penalty. This option must be used in combination with --lambda1 to ensure that sparsity is applied only at the individual feature level, not at the group (e.g., gene) level.
**--lambda1_grid**<br><float, float, float [0-1]>	Specifies a range of feature-level sparsity parameters (lambda1) for building multiple clade models, one model for each parameter. This option takes three float values: the starting value, the maximum value, and the step size. All values should be between 0 and 1. The parameter values increase linearly from the start to the maximum using the specified step, allowing exploration of different levels of feature sparsity.
**--lambda1_grid**<br><float, float, float [0-1]>	Specifies a range of feature-level sparsity parameters (lambda1) for building multiple clade models, one model for each parameter. This option takes three float values: the starting value, the maximum value, and the step size. All values should be between 0 and 1. The parameter values increase linearly from the start to the maximum using the specified step, allowing exploration of different levels of feature sparsity.
**--grid_rmse_cutoff**<br><float>	This option is to be used when lambda grids are used with multiple models. This helps to reduce the number of models to build and which models to use for aggregation.
**--grid_acc_cutoff**<br><float [0 1)>	Sets the minimum training accuracy threshold for retaining models during grid search. Any model with accuracy below this cutoff will be discarded.
**--grid_summary_only**	This option retains only the summary of all models generated during the grid search and discards the results of individual models.
**--min_group**<br><num>	Specifies the minimum number of groups that must be retained in each model during grid search. Models with fewer groups will be discarded.
**Data Preprocessing**	
**--include_singletons**	By default, MyESL removes all conserved sites and singleton variants

Option	Description
	(sites with only one differing sequence). Use this option to retain singleton sites during clade model construction if they are relevant to your analysis.
**--bit_ct**<br><num>	Excludes sites where mutations are observed fewer than <num> times across all sequences. This helps filter out extremely rare variants that may be uninformative or noisy.
**--data_type**<br><type>	Specifies the type of input sequence data MyESL will process.<br>Supported options are:<br>● nucleotide: For DNA sequences<br>● protein: For amino acid (AA) sequences<br>● molecular: For datasets containing both DNA and protein sequences<br>● universal: Allows any IUPAC characters, additional symbols, and numeric values (0–9), treating each unique character, including numbers, as distinct. This mode is case-sensitive, unlike the others, which are not.<br>Choose the appropriate type based on the format and diversity of your input data.
**Model Output**	
**--output**<br><name>	An output directory name can be defined by users. This directory will be created in the current working directory. If no output directory name is defined by users, a directory named "**output**" will be created by default.
**--gene_display_limit**<br><num>	Specifies the maximum number of gene groups (columns) to display in the model grid generated by MyESL. The default is 20, or fewer if the number of selected groups is less than 20.
**--species_display_limit**<br><num>	Specifies the maximum number of taxa (columns) from the clade of interest to display in the model grid generated by MyESL. The default is 20, or fewer if the number of selected taxa is less than 20.
**--gene_display_cutoff**<br><num>	Defines the number of genes or groups (columns) to display in the model grid. The default is 20, or fewer if fewer than 20 groups are selected.
**--m_grid**<br><row,col>	Sets the number of rows and columns in the model grid displayed by MyESL. The default is 20 for both dimensions, or fewer if the available rows or columns are less than 20.
**--stats_out**<br><PGHS>	Specifies the types of sparsity statistics to output from the model. Users can include any combination of the following:<br>**P:** Site-level (position) sparsity scores<br>**G**: Gene or group sparsity scores<br>**H**: Hypothesis sparsity score<br>**S**: Species prediction scores
**Miscellaneous**	
**--DrPhylo**	Enables DrPhylo analysis for a specified clade, either defined by clade ID

Option	Description
	using a phylogeny in a NEWICK format or via a response file in text format. DrPhylo builds multiple sparse models across combinations of site and group sparsity parameters. By default, it explores values from 0.1 to 0.9 (in steps of 0.1), generating 81 models. Users can customize this range using the --lambda_grid option. To reduce computation, DrPhylo skips models that include fewer than three genes. It outputs summary statistics, including PSS, GSS, and HSS scores, as well as a model grid. The default grid size is 20×20 (20 rows for species and 20 columns for genes), which can be adjusted using the --m_grid <row, col> option.
**--subsets**<br><int>	Enables subset-based analysis by dividing feature groups (e.g., genes) into multiple subsets. Models are trained on each subset, and informative groups are selected from each. A final model is then built using the combined set of selected groups. If --subsets 0, MyESL automatically determines the optimal number of subsets based on the system's computational capacity.
**Prediction**	
MyESL provides a separate command "**MyESL_model_apply.exe**" for applying the ESL model to predict traits or determine the clade membership of new species. To use this feature, users must provide sequence alignments that are aligned with the training alignments.	
<file name>	This positional input refers to the text file generated in the result directory of a MyESL analysis. The file, starting with "**MyES_model**", contains the ESL model, specifically, the estimated weights of the selected features.
<file name>	This is a positional option. In the second position, provide a text file listing the full paths to all sequence alignments that will be used for prediction.
**--response**<br><file name>	Specifies a text file where each line contains the name of a species for which clade membership or trait phenotype predictions will be performed.
**--output**<br><name>	Specifies the name of the directory where all results will be stored. If no name is provided, the output will be saved in a directory named "output" by default.
**--gene_display_limit**<br><int>	Sets the maximum number of genes to display in the grid image generated for the species specified with the --response option.
**--m_grid**<br><int,int>	Sets the number of rows and columns in the model grid displayed by MyESL. The default is 20 for both dimensions, or fewer if the available rows or columns are less than 20
